# 659. *In Vitro* Activity of Ceftazidime-Avibactam and Comparator Agents against *Enterobacterales* Collected from Patients with Bloodstream Infections (BSI) as Part of the ATLAS (India) Surveillance Program, 2019-2020

**DOI:** 10.1093/ofid/ofac492.711

**Published:** 2022-12-15

**Authors:** Abhisek Routray, Akshata Mane

**Affiliations:** Pfizer India Limited, Mumbai, Maharashtra, India; Pfizer India Limited, Mumbai, Maharashtra, India

## Abstract

**Background:**

Ceftazidime-avibactam (CAZ-AVI) is indicated for patients with bacteremia that occurs in association complicated intra-abdominal infection (cIAI), complicated urinary tract infection (cUTI) and hospital-acquired pneumonia (HAP). The present report evaluated the *in vitro* activity of CAZ-AVI and comparators against *Enterobacterales* clinical isolates collected from BSI as part of the ATLAS (The Antimicrobial Testing Leadership and Surveillance) surveillance program in 2019-2020 from India.

**Methods:**

A total of 2126 *Enterobacterales (493 from blood samples)* non-duplicate clinically significant isolates, were collected from 2019-2020. *In vitro* activity of Ceftazidime-avibactam and its comparators were assessed against *Enterobacterales* blood isolates.

**Results:**

Overall susceptibility to the β -lactam/β -lactamase inhibitor (BL-BLI) was 60 -76% for *Enterobacterales.* with highest susceptibility to Ceftazidime-avibactam. Overall highest susceptibility (97%) was noted for Tigecycline for *Enterobacterales*(n=476).The susceptibility to colistin was lower(83%) in *Enterobacterales* (n=409). Among 168 CR-*Enterobacterales* (CRE), Ceftazidime avibactam showed 29% susceptibility (n=49) while susceptibility to Tigecycline was 96%(n=161) followed by Colistin at 84%(n=141). Molecular analysis for CRE isolates showed 55 % (n=93) to be OXA-48 like producers (with or without NDM) ,68%(n=114)to be NDM producers (with or without OXA- 48) and 30%(n=51) to be co producers of OXA-48 like & NDM.

In vitro activity of commonly used antibiotics against Enterobacterales, blood isolates (2019-2020)

**Figure:**
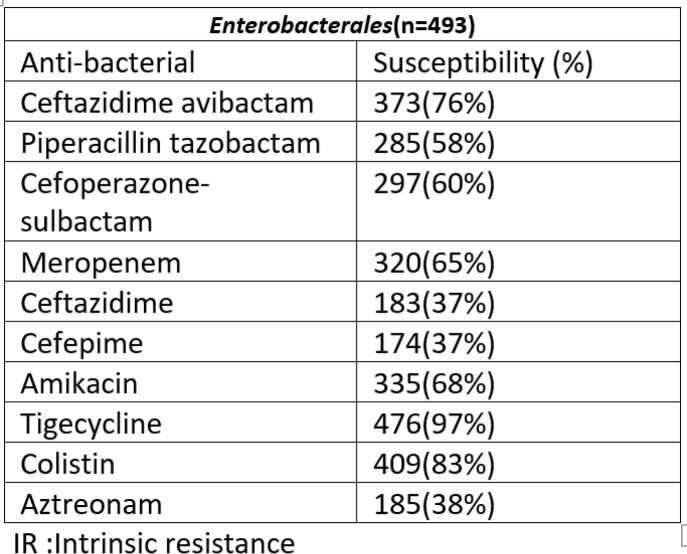

**Conclusion:**

Caz-Avi showed overall good susceptibility against the blood isolates tested hence it may be a valuable therapeutic option for treating BSI caused by MBL-negative *Enterobacterales.* Tigecycline followed by Colistin showed the highest susceptibilities. Tigecycline attains very low plasma concentrations and is not licensed for use in blood stream infections, while the use of colistin may be limited by the lack of CLSI susceptibility breakpoints and safety concerns.

**Disclosures:**

**Abhisek Routray, PhD**, Pfizer India Limited: Advisor/Consultant **Akshata Mane, MD**, Pfizer India Limited: Advisor/Consultant|Pfizer India Limited: Stocks/Bonds.

